# Regulation of Beclin 1-Mediated Autophagy by Oncogenic Tyrosine Kinases

**DOI:** 10.3390/ijms21239210

**Published:** 2020-12-03

**Authors:** Silvia Vega-Rubín-de-Celis, Lisa Kinch, Samuel Peña-Llopis

**Affiliations:** 1 Institute for Cell Biology (Cancer Research), University Hospital Essen, 45147 Essen, Germany; 2 Howard Hughes Medical Institute, University of Texas Southwestern Medical Center, Dallas, TX 75390, USA; lkinch@chop.swmed.edu; 3 Translational Genomics in Solid Tumors, German Cancer Consortium (DKTK) and German Cancer Research Center, University Hospital Essen, 45147 Essen, Germany; Samuel.Pena-Llopis@dkfz-heidelberg.de

**Keywords:** Beclin 1, autophagy, tyrosine kinases, cancer

## Abstract

Beclin 1 is a major regulator of autophagy, and it is a core component of the class III PI3K complexes. Beclin 1 is a highly conserved protein and its function is regulated in a number of ways, including post-translational modifications. Several studies indicate that receptor and non-receptor tyrosine kinases regulate autophagy activity in cancer, and some suggest the importance of Beclin 1 tyrosine phosphorylation in this process. Here we summarize the current knowledge of the mechanism whereby some oncogenic tyrosine kinases regulate autophagy through Beclin 1.

## 1. Introduction

Macroautophagy, herein referred to as autophagy (self-eating), is an intracellular degradation pathway whereby cytosolic components are engulfed into double-membrane structures (autophagosomes) for their degradation into the lysosome. Through this pathway, the cell can generate energy by recycling cytoplasmic components, but it can as well control cytoplasmic quality by degradation of damaged proteins and organelles, lipid droplets, or intracellular pathogens. Autophagy is an essential process involved in both physiological and pathological conditions, including protection against aging, infections, cancer and neurodegenerative, metabolic, inflammatory, and muscle diseases [1,2,3,4,5,6]. Beclin 1 is an essential autophagy protein, that is regulated through multiple post-translational modifications, including phosphorylation by oncogenic tyrosine kinases. Thus, the goal of this mini-review is to compile the data available regarding the autophagy regulation through Beclin 1 tyrosine phosphorylation in cancer.

## 2. The Role of Beclin 1 in Autophagy and Cancer

Beclin 1, the mammalian ortholog of the yeast Atg6/Vps30 is an evolutionary conserved protein that is essential for autophagy. Beclin 1 was discovered as a binding partner of Bcl-2 in a yeast-two hybrid screen [7], and it was shown that Bcl-2 and Bcl-X_L_ inhibit autophagy through their binding with Beclin 1 [8].

**Figure 1:**
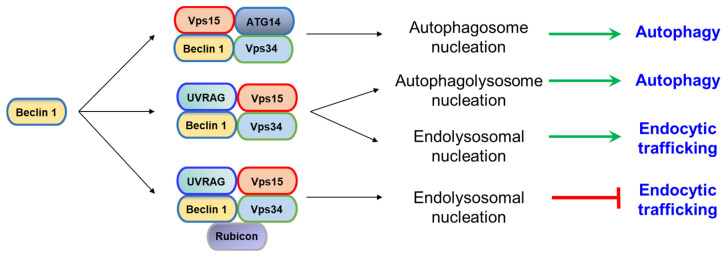
Beclin 1 forms several class III phosphatidylinositol 3-kinase (PI3KC3) complexes that regulate some steps of autophagy and endocytic trafficking.

Beclin 1 is part of two distinct mayor class III phosphatidylinositol 3-kinase (PI3KC3) complexes: PI3KC3-C1 (it is involved in autophagosome nucleation and it contains Beclin 1, VPS34, VPS15 and ATG14) and PI3KC3-C2 (it is implicated in endolysosomal and autophagolysosome nucleation and containing Beclin 1, VPS34, VPS15 and UVRAG). A third PI3KC3 has been described, in which Beclin 1 binds to VPS34, VPS15, UVRAG, and RUBICON to inhibit autophagic flux [9,10,11] (Figure 1).

One of the major events regulating autophagy is the formation of the Bcl-2/Bcl-xL—Beclin 1 complex formation. Bcl-2 and Bcl-xL inhibit autophagy through its binding to the BH3 domain of Beclin 1, stabilizing Beclin 1 homodimerization, disrupting Beclin 1 interaction with other components of the PI3KC3 complex and inhibiting autophagy [7]. The interaction of Bcl-2/Bcl-xL with Beclin 1 is regulated through multiple phosphorylation events that lead to promoting or disrupting the complex formation, including: (1) starvation-induced JNK1 (c-Jun amino terminal kinase 1) phosphorylation of Bcl-2 at T69, S70, and S97, promoting the dissociation of Bcl-2-Beclin 1 and the subsequent autophagy activation [12]; (2) Beclin 1 phosphorylation at T119 within its BH3 domain by DAPK (death associated protein kinase) or ROCK1 (Rho kinase 1) [13,14] promoting its dissociation from Bcl-2 and autophagy induction; (3) starvation-induced Beclin 1 phosphorylation at S90 by the stress responsive kinases MK2 and MK3, disrupting its binding with Bcl-2 and inducing autophagy [15]; (4) Mst1 (mammalian Ste20-like kinase 1) phosphorylation of Beclin 1 at T108 (within its BH3 domain), promoting the interaction of Bcl-2 and Beclin 1 and therefore inhibiting autophagy [16].

Beclin 1 (BECN1) is not frequently mutated in cancer. Only 106 out of 45,151 patients (less than 0.1%) from all tumor types found at cBioPortal (www.cbioportal.org) show alterations, and around 0.5% mutations (215 out of 38,262 unique samples) were found in the COSMIC database (Catalog of Somatic Mutations; https://cancer.sanger.ac.uk/cosmic). Beclin 1 functions as a haploinsufficient tumor suppressor, and allelic loss of Beclin 1 is frequently found in sporadic breast, ovarian, and prostate cancers [17,18,19,20]. Beclin 1 loss is associated with poor patient survival and more aggressive cancers [21] and mouse models harboring a single Beclin 1 copy have elevated incidence of spontaneous malignancies, including lung carcinomas, lymphomas, hepatocellular carcinomas and breast carcinomas [22,23,24].

## 3. Oncogenic Tyrosine Kinases

Tyrosine kinases (TKs) are enzymes that phosphorylate substrates at tyrosine residues and play essential roles in signal transduction, cell growth, migration, proliferation, differentiation, and survival. TKs are divided into receptor tyrosine kinases (RTKs) and non-receptor tyrosine kinases (NRTK).

### 3.1. Receptor Tyrosine Kinases

RTKs are transmembrane tyrosine kinases, they are classified in 20 different classes, and all of them share a common structure, having a highly glycosylated extracellular ligand binding domain, a single transmembrane domain, and an intracellular domain containing a tyrosine kinase domain and a carboxy-terminal tail [25]. Aberrant function of RTK lead to different diseases, including cancer. Under normal conditions, most RTK are activated by binding to their corresponding ligand through the extracellular domain, dimerization, and a conformational change resulting in *trans*-phosphorylation of each kinase and release of the *cis*-autoinhibition [26]. This phosphorylation and further activation recruit other molecules that activate downstream signaling pathways. Several mechanisms are involved into the aberrant activation of tyrosine kinases found in cancers, including gain-of-function mutations, increased RTK levels by overexpression or amplification, chromosomal rearrangements, kinase domain duplication, or autocrine activation [26,27]. Upon activation, RTK initiate a cascade of downstream signaling pathways, including the PI3K/Akt/mTOR pathway, MAPK, AMPK or the Ras/MEK/ERK pathway, and most of them are also implicated in autophagy regulation through different mechanisms [26].

### 3.2. Non-Receptor Tyrosine Kinases

NRTKs are cytosolic kinases with a quite variable structure subunit composition but that usually harbor a protein kinase domain and some protein–protein interaction domains, like Src Homology 2 (SH2), SH3 or PH (Pleckstrin homolog) domains, and are organized into 9 subfamilies [28]. NRTKs are involved in regulation of proliferation, cell growth, adhesion, migration, and apoptosis, and they also regulate signal transduction in the immune system [29]. Multiple oncogenic alterations have been described in NRTKs, including chromosomal rearrangements leading to fusion genes, typically associated with hematological malignancies [29].

## 4. Beclin 1 Regulation by Tyrosine Phosphorylation

Mammalian Beclin 1 function is regulated at different levels to modulate autophagy and other intracellular processes where Beclin 1 is involved, including vacuolar protein sorting or LC3-associated phagocytosis through the class III PI3Kinase complex. These multiple regulation levels include post-translational modifications (phosphorylation, ubiquitination, acetylation), changes in Beclin 1 subcellular localization (sequestration at the Golgi or at the cytoskeleton, or endosomal localization), or variations at the interactome.

Multiple Beclin 1 phosphorylation events have been reported that eventually modulate autophagy activity in mammals, either by induction or inhibition (reviewed in [30,31]), and multiple oncogenic kinases have also been involved in autophagy modulation through Beclin 1 phosphorylation [32]. Phosphorylation at Ser90 has been shown to activate autophagy through several kinases, including MK2/3 [15], DAPK3, [33], calcium-calmodulin-dependent protein kinase type II (CAMKII, [34]), AMP-activated protein kinase (AMPK, [35]), and also by the phosphatase PP2A [33]. Other activating phosphorylated residues in Beclin 1 include Ser93 and Thr388 (phosphorylated by AMPK, [36]), Ser30 (PGK1, [37]), and Thr119 (phosphorylated by DAPK [14] and ROCK1 [13]). Additional phosphorylation events in Beclin 1 were reported to inhibit autophagy in different ways, including (1) cytoskeletal sequestration of Beclin 1 through 14-3-3 binding upon AKT1 phosphorylation at residues Ser234 and Ser295 [38]; (2) Beclin 1 dimerization and modification of its binding partners upon phosphorylation at Tyr229, Tyr233 and Tyr352 by EGFR [39] or at Tyr233 by HER2 mutants [40] and FAK [41]; (3) Increased binding of Beclin 1 with Bcl-2 and decreased binding with Vps34 through Beclin 1 phosphorylation at Thr108 by Mst-1 [16].

**Figure 2:**
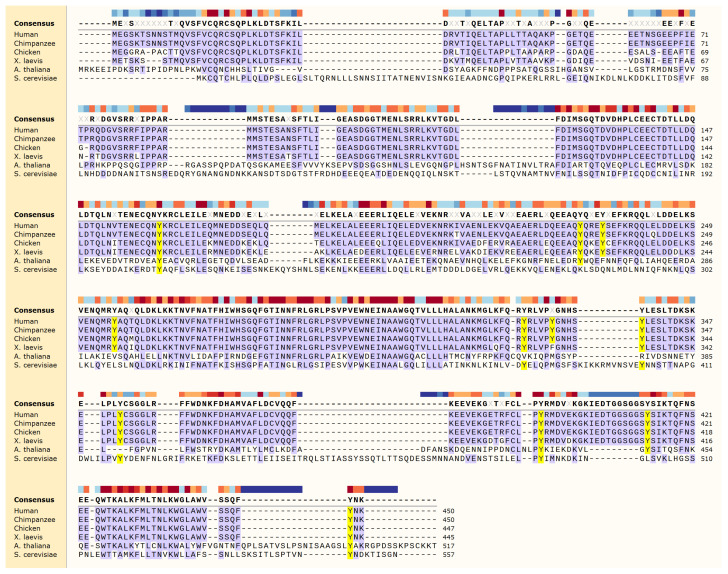
Beclin 1 protein sequence alignment. Tyrosine residues are highlighted in yellow. Color-coded bar at the top indicated conservation degree. Alignment was generated using Multiple Sequence Comparison by Log-Expectation (MUSCLE) algorithm in SnapGene (GSL Biotech).

**Figure 3:**
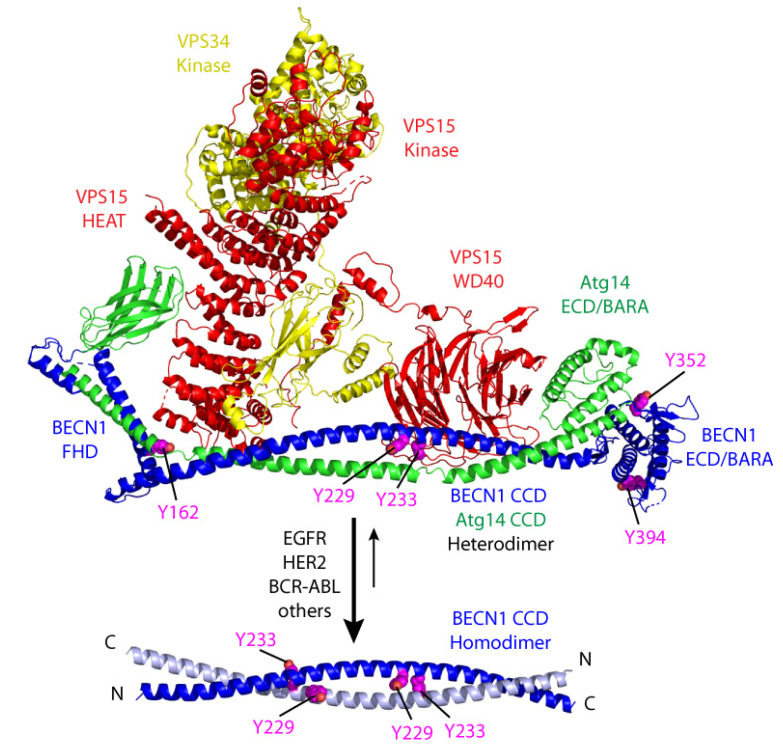
Model of tyrosine phosphorylation modulating the equilibrium between Beclin 1 active CCD heterodimer and inactive CCD homodimer. Human VPS34 complex model above is based on the yeast structure (PDB:5dfz). Complex subunits are colored: VPS15 (PIK3R4) in red, VPS34 (PIK3C3) in yellow, ATG14 in green and Beclin 1 in blue, with Beclin 1 Tyr sidechains in magenta sphere. The heterodimeric Beclin 1-ATG14 CCD cradles the VPS15/VPS34 heterodimer in the complex, with the Y233 sidechain pointing towards the VPS15 beta-propeller, suggesting the phosphorylation state would influence complex formation. Alternately, the interaction of the Beclin 1 CCD homodimer below (PDB: 5hhe) competes with complex formation. Tyrosine phosphorylation shift the equilibrium to the homodimeric state and inhibit autophagy.

Human Beclin 1 protein contains a total of 11 tyrosines: Y162 (flexible helix domain, FHD), Y229, Y233, Y256 (CCD domain) and Y328, 333, 338, 352, 394, 413, and 448 located within the C-terminal ECD-BARA domain (Figure 2). Some of them are highly conserved across species, from human to yeast, including Y162 (located at the FHD domain) and Y394 and Y448 (located within the BARA domain). In the structure of the yeast VPS34 complex II [42], the FHD helix interacts with a helix from the VPS38 (UVRAG) and a helix from the scaffolding ARM repeat of VPS15 (PI3R4) (Figure 3). The Y162 sidechain points towards the intersection of these two helices, suggesting it may play a role in positioning the complex. The Y394 sidechain is exposed to solvent in the complex structure model, suggesting that phosphorylation would be allowed in the complex (Figure 3). So far, only a handful of these residues have been shown to be phosphorylated by tyrosine kinases, and they all have an effect on regulating autophagy.

A crystal structure of the yeast VPS34 complex II [42] includes VPS34 (PIK3C3), VPS15 (PIK3R4), VPS30 (BECN1), and VPS38 (ATG14 or UVRAG). This structure provides a model for Beclin 1 interaction with the human complex components and its activation of phosphatidylinositol-3-kinase (PI3K) that leads to autophagy. The central coiled coils (CCD) of Beclin 1 and ATG14 (or the structurally related UVRAG of complex II) form a parallel heterodimer, which positions the Beclin 1 ECD/BARA domain at the tip of one arm of a Y-shaped complex structure (Figure 3).

The Beclin 1/ATG14 heterodimeric CCD forms an elongated platform for its interaction with an intertwined VPS15/VPS34 kinase heterodimer. The interaction positions the active site of the VPS34 kinase at the tip of the other arm of the Y. The presence of the Beclin 1 CCD heterodimer increases PI3K activity on vesicles. Deletion of a BARA domain “aromatic finger”, which is thought to mediate interaction with the membrane, lowers this activity [42,43]. Thus, activation of PI3K by the Beclin 1 CCD heterodimer is thought to increase VPS34 PI3K activity by interacting directly with the membrane where substrate resides.

In the absence of ATG14, the Beclin 1 CCD alone forms metastable antiparallel homodimers that transition to heterodimeric CCD upon addition of ATG14 [44,45]. The Beclin 1 homodimeric state is mimicked by the full-length protein, with the BARA domain contributing to the strength of the CCD interaction [46], suggesting that the CCD equilibrium is influenced by other Beclin 1 domains. Thus, phosphorylation of Beclin 1 could modulate the equilibrium between its inactive homodimeric CCD state and the active heterodimeric CCD that allows complex formation (Figure 3).

## 5. Beclin 1 Phosphorylation by Receptor Tyrosine Kinases

### 5.1. EGFR

Epidermal growth factor receptor (EGFR) belongs to the class I of the ERBB receptor tyrosine kinases. Several ligands have been shown to bind EGFR, including TGFA (transforming growth factor-alpha), heparin-binding EGF-like growth factor (HBEGF) and betacellulin (BTC), although EGF is the most studied of all. Upon binding to its ligand, EGFR homo or heterodimerize with other family members (including HER2, HER3, HER4), leading to autophosphorylation and further recruitment of other partner proteins for intracellular signal transduction, activating the Ras/MAPK pathway, PI3K/Akt pathway, and STAT pathway. EGFR signaling is aberrantly activated in a number of cancers, including lung, head and neck, colon, brain, and pancreas, due to activating mutations, amplifications, or increased protein levels [47,48,49,50].

**Figure 4:**
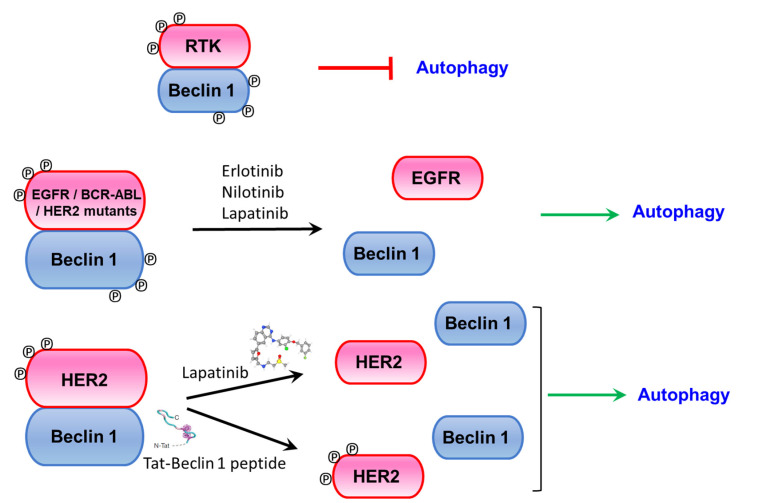
Models on RTK-Beclin 1 complex effects and autophagy induction upon receptor tyrosine kinase inhibitor or Tat-Beclin 1 autophagy-inducing peptide treatment.

EGFR was shown to be an autophagy modulator through regulation of Beclin 1 tyrosine phosphorylation. Previous studies had shown that EGFR inhibitors treatment induced autophagy in multiple cancer cell lines [51,52], but the specific mechanism underlying this process was unknown. Wei and colleagues [39] showed that activated EGFR upon ligand binding promote Beclin 1-EGFR interaction at the endosomes and further Beclin 1 phosphorylation at tyrosine residues 229, 233, and Y352. These phosphorylation events promote the formation of Beclin 1 homodimers, since phosphorylated Y229/Y233 stabilize the Beclin 1 CCD homodimer, losing the ATG14 or UVRAG CCD interaction and therefore blocking the binding of the VPS34 kinase (Figure 3). Thus, tyrosine phosphorylation by EGFR releases Beclin 1 interaction with ‘activating’ binding partners such as UVRAG, VPS34, ATG14, or VPS15 and promotes Beclin 1 homodimerization and binding to inhibitory proteins such as Bcl-2 and Rubicon, leading to autophagy inhibition. Interestingly, this effect is independent of the mTORC1 (mammalian target of rapamycin complex 1) activity, a well-known regulator of autophagy activity and a downstream target of EGFR. The importance of EGFR-phosphorylated Beclin 1 in tumorigenesis is highlighted by xenograft experiments, where NSCLC (non-small cell lung cancer) cells expressing a constitutively phosphorylated Beclin 1 mutant inhibit autophagy and enhance tumor growth and proliferation [39]. Interestingly, treatment with the receptor tyrosine kinase inhibitor Erlotinib abolished EGFR and Beclin 1 phosphorylation, disrupted EGFR/Beclin 1 binding and induced autophagy (Figure 4).

A role of inactive EGFR on regulating autophagy to promote cell survival under serum starvation or stress conditions was also described. Inactive EGFR binds to LAPTM4B (lysosomal protein transmembrane 4b) and Sec5 (EXOC2, exocyst complex component 2) and competes with Beclin 1 for its binding with Rubicon, activating autophagy [53].

### 5.2. HER2

HER2 (ERBB2, v-erb-b2 avian erythroblastic leukemia viral oncogene homologue 2) is an oncogenic receptor tyrosine kinase of the EGFR family. It is amplified in around 20–25% of breast cancers and other cancer entities—such as esophagus, bladder, and cervical cancer [54,55]—and such amplification correlates with poorer prognosis. HER2 somatic mutations are also found in several cancers—such as breast, small bowel, lung, cervical, bladder, and non-melanoma tumors—mostly in tumors with no *HER2* gene amplification. Such mutations are mostly missense mutations found either within the tyrosine kinase and the extracellular domains or insertions in exon 20 [54]. HER2 is an orphan receptor, and amplifications lead to increased protein production and activation through homo- and hetero-dimerization with other family members, and preferentially with HER3 and HER4 [56,57]. Upon activation, it initiates multiple intracellular cascade pathways, like the mitogenic-activated protein kinase (MAPK), RAS/MEK/ERK, PI3K and STAT, promoting cell proliferation and survival. Current treatments for HER2+ breast cancer include receptor tyrosine kinase inhibitors (Lapatinib and Afatinib, that target both EGFR and HER2; Neratinib, that only binds to HER2), which block intracellular kinase activity, antibodies targeting the extracellular domain and inhibiting HER2 dimerization (Trastuzumab, Pertuzumab) or antibodies combined with a microtubule de-polymerization agent (Trastuzumab Emtansine; T-DM1). An antibody against p95HER2, an active C-terminal fragment of HER2, found in 40% of HER2 positive tumors have also been investigated as a potential therapy for this subset of patients [58].

Some studies suggested an association between Beclin 1 and HER2 in breast cancer, and a correlation between HER2 amplification in breast cancer with *BECN1* DNA copy loss was found [59]. Furthermore, low *BECN1* mRNA expression was associated with HER2 amplification [60] and a much poorer disease-specific survival in HER2+ breast cancer [21]. In vitro analysis of breast cancer cells overexpressing HER2 compared to other HER2- cell lines also suggested an inhibitory effect of HER2 on autophagy [60,61]. These data indicate a potential role of autophagy in HER2+ breast tumorigenesis, although the specific relationship between autophagy, and particularly Beclin 1 and HER2 was not addressed. Later on, it was discovered that HER2 binds to Beclin 1 in HER2+ tumor cells [40,62] and inhibits autophagy. Although such binding and the corresponding effects on autophagy inhibition are dependent on HER2 kinase activity (a kinase dead mutant D845A does not bind to Beclin 1 and fails to inhibit autophagy), despite its binding, wild-type overexpressed HER2 does not appear to phosphorylate Beclin 1 at least at levels detectable by conventional techniques. The mechanism underlying this effect is unclear, but treatment of HER2+ breast cancer cell lines with the tyrosine kinase inhibitor Lapatinib disrupts the HER2-Beclin 1 complex and induces autophagy [40,62]. Overexpression of an activated HER2 mutant (A775_G776insYVMA), however, phosphorylate Beclin 1 at Y233, also leading to autophagy inhibition. Thus, even though several HER2 forms bind to Beclin 1, wild-type HER2 does not modulate autophagy through direct Beclin 1 phosphorylation, but rather through a different, currently unclear, mechanism. Studies related to Alzheimer’s disease suggest that in this setting HER2 binding to Beclin 1 might compete with the recruitment of other proteins, including VPS34 and VPS15, therefore inhibiting autophagy [63]. However, the full details underlying this mechanism remain elusive. Thus, HER2 inhibits autophagy at least through two different mechanisms: (1) Overexpressed wild-type HER2 binds to Beclin 1 and inhibits autophagy in an mTORC1-dependent and Beclin 1-tyrosine phosphorylation independent manner; (2) Activating HER2 mutants phosphorylate Beclin 1 at Y233, promoting Beclin 1 homodimerization and inhibition of autophagy in an mTORC1-independent manner (Figure 3).

HER2+ breast cancer cells as well as xenografts derived from them are able to induce autophagy upon Lapatinib treatment, and this correlates with inhibition of HER2 phosphorylation, disruption of the HER/Beclin 1 complex and induction of autophagy. These effects might be due, at least in part, to the inhibition of other HER2/EGFR downstream pathways, and more targeted and specific treatments with an autophagy inducing peptide were tested. Tat-Beclin 1 peptide promotes autophagy through releasing Beclin 1 from its inhibitory partner GAPRA1 [64] and therefore requires Beclin 1 and downstream autophagy machinery to induce autophagy. It contains 11 amino acids from the evolutionarily conserved region of Beclin 1 [65] and it induces autophagy in vitro in multiple cell lines. It is well tolerated and also induces autophagy in vivo where has been shown to protect from infection [64,66], cardiac disease [67,68], bone disease [69], and axonal injury [70]. Tat-Beclin 1 treatment compromised the growth of xenografts derived from HER2+ breast cell lines at a similar extent as Lapatinib treatment [40]. Furthermore, it disrupted the Beclin 1/HER2 complex, activated autophagy without changes in the HER2 phosphorylation status and induced a transcriptional signature different from the one in Lapatinib-treated tumors (Figure 4). Taken together, these data indicate that autophagy plays a major role in regulating HER2+ tumor growth. HER2 overexpression found in other cancers, like esophageal adenocarcinoma, was shown to also have an inhibitory effect on autophagy, and treatment with the dual EGFR/HER2 inhibitor Lapatinib also induced autophagic flux in vitro [71].

It was demonstrated that autophagy is also essential for tumor development in HER2+ breast cancer. Transgenic mice overexpressing HER2 under the control of an MMTV mammary-specific promoter (FVB/N-Tg MMTVneu) were crossed with mice harboring a whole-body knock-in mutation (*Becn 1*^F121A/F121A^) that releases the Bcl-2 inhibitory effect on Beclin 1 [72] and therefore show increased basal autophagy activity in multiple tissues, including the mammary gland. None of the *Becn 1*^F121A/F121A^ mice developed tumors by 450 days of life, whereas around 25% of the *Becn 1*^WT/WT^ or the *Becn 1*^WT/F121A^ developed mammary tumors [40]. These data demonstrate that bypassing the HER2 effects on Beclin 1 and autophagy by increasing basal autophagy blocks HER2-mediated tumorigenesis *in vivo*. Although *Becn1^+/−^* mice show an increased susceptibility to tumor formation and an elevated incidence of multiple malignancies [23,24] crossings of *Becn1^+/−^* mice with mouse models of *Erbb2*- or *PyMT*-driven mammary tumorigenesis had no effect on tumor development ([60]; Vega-Rubín-de-Celis, unpublished data).

## 6. Beclin 1 Phosphorylation by Non-Receptor Tyrosine Kinases

### BCR-ABL

Fusion kinase BCR-ABL results from a fusion between the Breakpoint cluster region (BCR) on chromosome 22 and the Abelson murine leukemia viral oncogene homolog 1 (ABL) located in chromosome 9 [73]. It is found in ~90% of patients with chronic myeloid leukemia (CML) and ~20–30% of patients of acute lymphoblastic leukemia (ALL) and encodes for the so called “Philadelphia chromosome” (Ph). BCR-ABL forms homodimers through the BCR coiled-coil domain, rendering the ABL kinase constitutively active, and further activating several downstream pathways implicated in cell growth and proliferation, including the MAPK, CRKL, GRB2/GAB2, PI3K/Akt, and JAK/STAT pathways [74,75]. Over the years, several BCR-ABL inhibitors have reached the clinic for treatment, including Imatinib and Nilotinib (ATP-binding competitors; [76,77]), Dasatinib and Bosutinib (dual SRC/ABL1 inhibitors; [78,79]) or Asciminib (ABL1 allosteric inhibitor; [80]).

Some reports implicated autophagy as a potential target in Ph+ leukemias [81,82,83,84,85,86,87], and a recent paper highlighted the role of Beclin 1 phosphorylation in autophagy regulation by BCR-ABL [88]. In vitro experiments of Beclin 1 depletion through miRNA in Ba/F3 cells indicated a role of Beclin 1 in cell proliferation and apoptosis, and in vivo transplantation analysis showed a prolonged survival on Beclin 1 knock-down BCR-ABL+ BMDC compared to control samples [88]. Interestingly, these effects might be autophagy-independent, since similar experiments depleting another autophagy essential gene, Atg5, had no effect on survival. Further research into the mechanism underlying the role of Beclin 1 in leukemia lead to the finding that BCR-ABL binds to Beclin 1 and phosphorylates it at Y233 and Y352. These phosphorylation events lead to autophagy inhibition. Nilotinib treatment compromised the Beclin 1/BCR-ABL binding (Figure 3), and induced autophagy in a way that is dependent on Beclin 1 tyrosine phosphorylation, since Beclin 1 phosphomimetic mutant Y233/352E fail to induce autophagy upon RTK inhibitor treatment. The specific function of these Beclin 1 tyrosine residues in vivo and whether the effects on survival are autophagy-dependent remain to be determined.

## 7. Other Tyrosine Kinases Regulating Autophagy

Other oncogenic tyrosine kinases have been involved in regulating autophagy, especially since treatments with tyrosine kinase inhibitors in many cases induce autophagy. However, it is still unclear whether these processes involve Beclin 1 tyrosine phosphorylation or there are other mechanisms implicated. For instance, Rearranged during transfection (*RET*), a Class XIV Receptor Tyrosine Kinase is associated with multiple malignancies through different mechanisms, including activating mutations and gene fusions [89,90,91,92,93,94,95], and it was recently identified in a shRNA screen as an essential gene in AML that activates mTORC1 and therefore inhibits autophagy [96]. Conversely, *RET* knockdown induced autophagy and a correlation between high RET and high p62 levels was found in patients [96]. The role of amplified fibroblast growth factor receptor 1 (FGFR1) in autophagy in NSCLC was also explored in a recent study where it was reported that FGFR1 activation by its ligand inhibit autophagy through the ERK-MAPK pathway and regulation of the total Beclin 1 levels [97]. A recent report [98] described discoidin domain receptor 1 (DDR1) as an autophagy regulator in glioblastoma resistance to radiochemotherapy through its binding to the 14-3-3/Akt/Beclin 1 complex [38]. DDR1 inhibition induces autophagy and sensitizes cells to radiation in a way that is Beclin 1-dependent. Anaplastic lymphoma kinase (ALK) is a receptor tyrosine kinase of the Class XVI that has been found to be implicated in multiple cancers through aberrant activation due to point mutations, overexpression, or a diversity of translocations giving rise to fusion proteins [99]. Studies in ALK+ anaplastic large cell lymphoma showed that treatment with the tyrosine kinase inhibitor crizotinib induced autophagy and that combination of ALK inhibition with Bcl-2 depletion induced autophagy and cell death [100,101], and autophagy induction was also found upon ALK inhibition in glioblastoma [102]. Aberrant RTK signaling is also involved in hepatocellular carcinoma (HCC), and RTK inhibitor sorafenib is used for therapy and was shown to induce autophagy in multiple systems [103].

## 8. Conclusions

Autophagy is regulated by tyrosine kinases directly or indirectly in multiple ways, through mechanisms largely unexplored. Some of them involve the phosphorylation of Beclin 1 at different tyrosine residues that ultimately regulate the activity of the PI3KC3 and affect tumor growth. Further and deeper studies are required to delineate the fine-tuned mechanism in each particular context and tumor type.
